# Plexins function in epithelial repair in both *Drosophila* and zebrafish

**DOI:** 10.1038/ncomms12282

**Published:** 2016-07-25

**Authors:** Sa Kan Yoo, Heath G. Pascoe, Telmo Pereira, Shu Kondo, Antonio Jacinto, Xuewu Zhang, Iswar K. Hariharan

**Affiliations:** 1 Department of Molecular and Cell Biology, University of California, Berkeley, California 94720, USA; 2 The Miller Institute, University of California, Berkeley, California 94720, USA; 3 Physiological Genetics Laboratory, RIKEN, 2-2-3 Minatojima-minamimachi, Chuo-ku, Kobe 650-0047, Japan; 4 Department of Pharmacology, University of Texas Southwestern Medical Center, Dallas, Texas 75390, USA; 5 Instituto de Medicina Molecular, Faculdade de Medicina da Universidade de Lisboa, 1649-028 Lisboa, Portugal; 6 CEDOC, Chronic Diseases Research Centre, NOVA Medical School, Universidade NOVA de Lisboa, 130, 1169-056 Lisboa, Portugal; 7 Genetic Strains Research Center, National Institute of Genetics, 1111 Yata, Mishima, Shizuoka 411-8540, Japan

## Abstract

In most multicellular organisms, homeostasis is contingent upon maintaining epithelial integrity. When unanticipated insults breach epithelial barriers, dormant programmes of tissue repair are immediately activated. However, many of the mechanisms that repair damaged epithelia remain poorly characterized. Here we describe a role for Plexin A (PlexA), a protein with particularly well-characterized roles in axonal pathfinding, in the healing of damaged epithelia in *Drosophila*. Semaphorins, which are PlexA ligands, also regulate tissue repair. We show that *Drosophila* PlexA has GAP activity for the Rap1 GTPase, which is known to regulate the stability of adherens junctions. Our observations suggest that the inhibition of Rap1 activity by PlexA in damaged *Drosophila* epithelia allows epithelial remodelling, thus facilitating wound repair. We also demonstrate a role for Plexin A1, a zebrafish orthologue of *Drosophila* PlexA, in epithelial repair in zebrafish tail fins. Thus, plexins function in epithelial wound healing in diverse taxa.

## Abstract (web summary)

Plexins are semaphorin receptors and are well known for their roles in neuronal pathfinding. Here the authors describe a role for Plexin A in healing damaged epithelia in *Drosophila* and zebrafish. In *Drosophila*, Plexin A inhibits the GTPase Rap1 to allow epithelial remodelling to facilitate wound repair.

## Graphical abstract

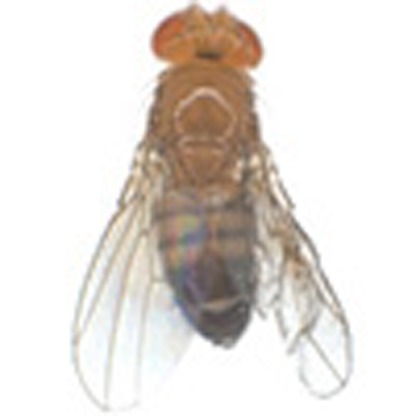

The initial responses to epithelial wounds have an important role in determining whether tissue damage eventually results in regeneration or scar formation^1^,^2^,^3^. Since epithelial tissues are found in most metazoans, some of the mechanisms involved in repairing epithelial tissues and in healing wounds are likely to be evolutionarily ancient and can potentially be identified using genetic screens conducted in invertebrate model organisms^4^,^5^,^6^,^7^. We first screened for regulators of wound repair in *Drosophila* imaginal discs and then tested whether the orthologous genes in zebrafish have a similar function. Using this approach, we have identified a key role for plexins during tissue repair in both organisms.

## Results

### An RNAi screen identifies a role for *PlexA* in wing disc repair

**Figure 1:**
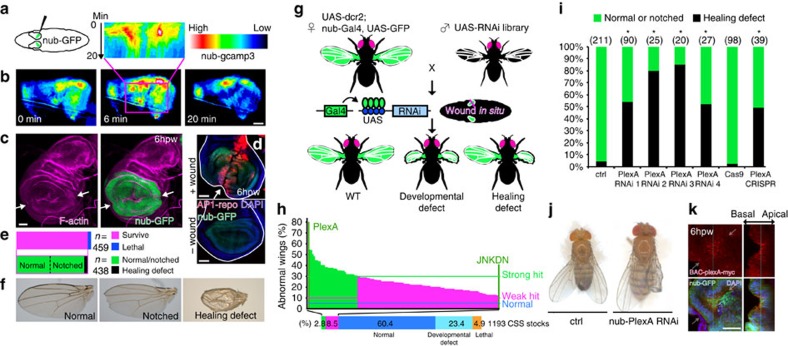
An RNAi screen identifies a role for *PlexA* in wing disc repair. (**a**) Diagram of *in situ* wounding of wing discs (Supplementary Movie 1). (**b**) Damage induces a transient calcium flash in wing discs (Supplementary Movie 2). The top inset is a kymograph derived from the region shown in the rectangular box. (**c**,**d**) F-actin accumulates and the AP-1 reporter is activated at the wound edges (white arrows), which have fused by 6 h post wounding (h.p.w.). (**e**,**f**) Adult wing phenotypes after wounding wing discs of L3 larvae. (**g**,**h**) Scheme of the wing disc-specific RNAi screen and classes of phenotypes obtained. Overall, 1,193 CSS RNAi lines were used. (**i**,**j**) Four independent *PlexA* RNAi transgenes and the wing disc-specific *PlexA* knockout with CRISPR induce healing defects. *P<0.05, two-tailed *χ*^2^-test with the Bonferroni correction (RNAi), two-tailed *χ*^2^-test (CRISPR). (**k**) PlexA expression in wounded wing discs of *PlexA-myc* BAC transgenic flies. Arrows indicate the wound location. Scale bars, 50 μm.

The adult wing of *Drosophila* develops from a larval epithelial structure, the wing imaginal disc that can be visualized *in situ* by the *nubbin>GFP* (green fluorescent protein) reporter and bisected by applying pressure on the larval cuticle with a tungsten needle while leaving the cuticle itself intact (Fig. 1a and Supplementary Movie 1). After wounding, the two portions of the wing disc were observed to move independently but remained in proximity to each other likely because they were attached to neighbouring structures. To examine early changes following wounding, we used a GCaMP3 probe to visualize changes in intracellular calcium^8^. In the vicinity of the wound, we observed a rapid increase immediately after wounding, which persisted for 5–10 min (Fig. 1b and Supplementary Movie 2), which is similar to the wound-induced calcium flashes observed in the *Caenorhabditis. elegans* epidermis^9^, the zebrafish tail fin^2^ and the *Drosophila* embryonic and pupal epithelia^10^,^11^. Fusion of the two disc fragments had occurred within 6 h of wounding. At this time, there was an accumulation of F-actin at the site of fusion (Fig. 1c) and increased activity of the Jun N-terminal kinase (JNK) as assessed by a transcriptional reporter of AP-1 activity^12^ (Fig. 1d). Calcium signalling, accumulation of F-actin and the increased activity of JNK are likely to be evolutionarily ancient responses to wounding since they are found in diverse organisms. Thus, screens for regulators of wound healing in imaginal discs are likely to identify mechanisms that are conserved among diverse taxa.

Approximately 95% of flies survive when one of their wing discs has been bisected (Fig. 1e,f). In these survivors, rejoining of the two fragments is highly efficient and ∼95% of the discs develop into adult wings that either look completely normal or have minor notching of the wing margin. In the remaining 5% of the adult survivors, there are a variety of defects, most notably blisters, which are often seen when the two surfaces of the adult wing fail to adhere properly.

To test whether a gene has a role in wound healing, we manipulated its activity in the wing pouch and fragmented one of the two wing discs; the intact contralateral disc served as a control (Fig. 1g). A dominant-negative form of the JNK (JNK^DN^) elicited an increase in the frequency of wing morphology defects on the wounded side (43% versus 5% in controls; Fig. 1h) consistent with previous observations in other systems that indicated a role for JNK in wound healing^4^,^5^,^13^,^14^,^15^. We then screened 1,193 RNA interference (RNAi) lines targeting proteins that are predicted to be either on the cell surface or secreted (CSS)^16^ because these CSS molecules are likely to play important roles in cell–cell communication and tissue remodelling during wound repair. We wounded at least 25 larvae per stock, thus screening ∼30,000 flies (Fig. 1h). Of these, 34 lines (2.8%) were classified as ‘strong hits' since they elicited wing abnormalities only on the wounded side. *Plexin A* (*PlexA*) was the top hit, with ∼80% of wounded animals exhibiting wing abnormalities with only minor effects in the absence of wounding, a result confirmed using three additional RNAi lines (Fig. 1i,j and Supplementary Fig. 1a,b). In addition, to inactivate *PlexA* function in the wing disc in a way that does not depend on RNAi, we expressed the Cas9 nuclease and a guide RNA (gRNA) that targeted *PlexA* in cells of the wing pouch. Under these conditions, as with RNAi lines that targeted *PlexA*, proper repair was not observed (Fig. 1i and Supplementary Fig. 1c). *PlexA* is indeed expressed in wing discs, and its expression was more pronounced around wound edges (Fig. 1k and Supplementary Fig. 1d–f).

### *PlexA* regulates wound closure in the epithelium of the notum

**Figure 2:**
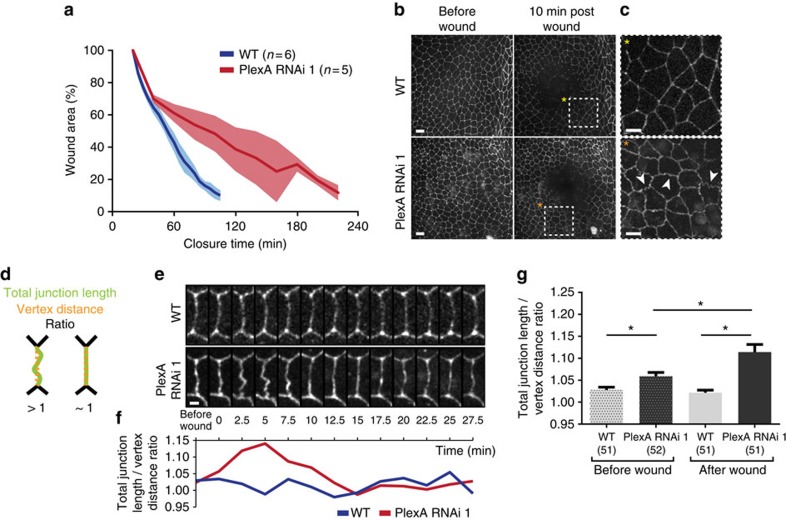
PlexA regulates wound closure in the epithelium of the notum. (**a**) *PlexA* RNAi slows down wound closure upon laser wounding in the pupal notum at 13 APF. Shadowed areas represent s.d. (**b**,**c**) Expression of E-Cad-GFP in notum epithelia. Cells in the *PlexA* RNAi epithelium exhibit wiggly cell boundaries around the wound (indicated with white arrowheads) when compared with controls. Scale bars, (**b**) 10 μm and (**c**) 5 μm. (**d**) Schematic diagram of the ratio used to quantify the membrane defects. (**e**,**f**) Quantification of the junction length and vertex distance ratio over time in controls and *PlexA* RNAi. The membrane morphology change reaches its maximum at 5 min after wounding. Scale bar, 2 μm. (**g**) Junction length and vertex distance ratio for controls and *PlexA* RNAi both before and after wound for five independent pupae for each condition. Error bars represent s.e.m. *P<0.05; Kruskal–Wallis test with Dunn's post-test.

In order to directly visualize the effect of *PlexA* inhibition on the dynamics of wound closure, we examined the closure of wounds generated by a laser in the epithelium of the pupal notum, which is amenable to live imaging. In control pupae, laser-generated wounds closed within 120 min (Fig. 2a and Supplementary Movie 3). In contrast, expression of a *PlexA* RNAi resulted in a delay in wound closure, taking 180–240 min. In the pupal notum, it was also evident that the *PlexA* RNAi made the cell boundaries, which were visualized using E-cadherin–GFP, more wiggly in the region around the wound (Fig. 2b,c). To better analyse this phenomenon, we measured the ratio between the total junction length and the distance between the correspondent vertexes for each condition (Fig. 2d). This ratio fluctuates along time in both control and *PlexA* RNAi (Fig. 2e,f), but in the latter there is a peak of the ratio ∼5 min post wounding, when the changes in cell outlines are more pronounced. Using this time point as a post-wounding reference we measured this ratio using cell outlines from five independent pupae for each condition, both before and after wounding (Fig. 2g). With this analysis we observed that even before wounding, *PlexA* inhibition is sufficient to make the junction morphology more wiggly when compared with control, but also that when the tissue is challenged by a laser wound this phenotype is significantly more pronounced. Wiggly cell outlines have been previously observed when cell–cell adhesion is altered^17^,^18^. Thus, in the epithelium of the notum, *PlexA* function is necessary for efficient wound closure and a reduction in *PlexA* function likely results in alterations in cell adhesion.

### PlexA signalling regulates wound repair

**Figure 3:**
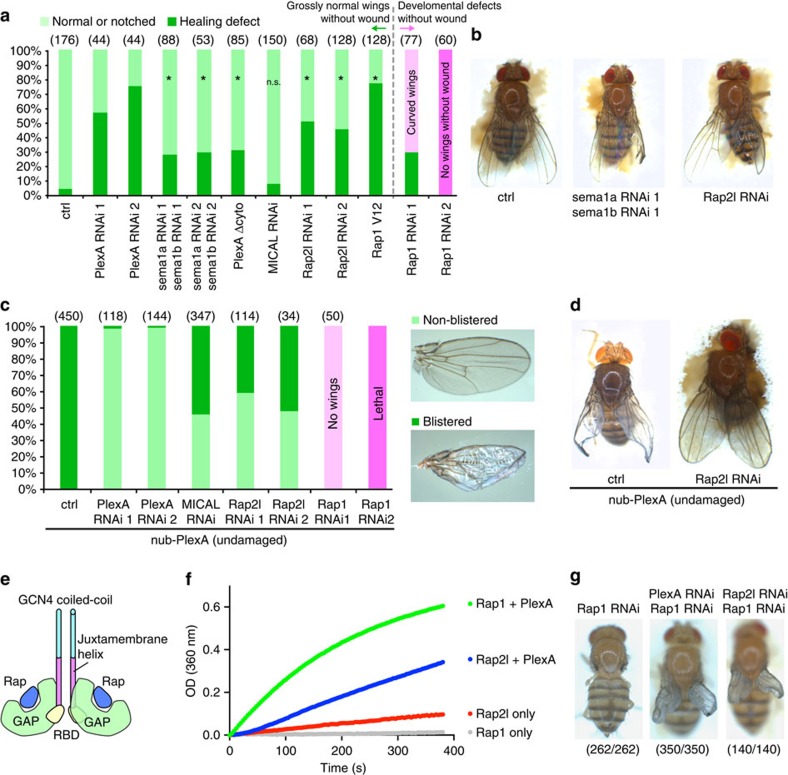
PlexA signaling regulates wound repair. (**a**,**b**) Double knockdown of *sema1a* and *sema1b*, expression of PlexA Δcyto, knockdown of *Rap2l* or expression of constitutively active Rap1 V12 perturbs wound healing but *MICAL* knockdown does not. Note that only right wings are wounded. The flies were held in a hole of a sponge, which looks brown beneath flies, for image acquisition. *P<0.05, two-tailed *χ*^2^-test with the Bonferroni correction. (**c**,**d**) Ectopic expression of *PlexA* in the wing pouch induces blisters in adult wings. This effect is prevented by *Rap2l* or *MICAL* knockdown and enhanced by *Rap1* knockdown. (**e**) Schematic diagram of the coiled-coil PlexA_cyto_ fusion protein to enable constitutive dimerization. (**f**) The Rap GAP activity of Coiled-coil PlexA_cyto_ was measured using the photometric assay. The slope of the linear portion of the reaction curves reflects the initial rate of GTP hydrolysis (see Methods for details). The rate of GTP hydrolysis of Rap1 catalysed by PlexA (0.166 O.D. per min) is approximately ninefold higher than that of Rap2l (0.019 O.D. per min). (**g**) *Rap1* knockdown-induced loss of adult wings is suppressed by inhibition of *PlexA* or *Rap2l*.

The role of plexins has been best characterized in the context of axonal pathfinding^19^,^20^,^21^,^22^. Plexins are transmembrane proteins that bind to their ligands, semaphorins, and then regulate the actin cytoskeleton to mediate neurite repulsion^19^,^20^,^21^,^23^. The flavoprotein oxidase Mical has been shown to function downstream of plexins in this process. To determine whether these other components of the semaphorin/plexin pathway are also involved in tissue repair in imaginal discs, we first tested whether they were expressed in imaginal discs. Expression of all the known plexins, semaphorins and *Mical* was detected in discs by reverse transcriptase PCR (RT–PCR; Supplementary Fig. 2a–c). We then tested the requirement of Sema1a and Sema1b, which are ligands for PlexA^19^,^20^,^21^,^22^, in the repair of damaged discs. While knockdown of either *Sema1a* or *Sema1b* had little effect (Supplementary Fig. 2d), simultaneous knockdown of both *Sema1a* and *Sema1b* increased the frequency of adult wings of abnormal morphology on the wounded side (Fig. 3a,b). The weaker effect of knockdown of *Sema1a* and *Sema1b* when compared with *PlexA* could be because of either partial knockdown or redundancy with other semaphorins. Overexpression of a truncated form of PlexA that lacks a cytoplasmic domain (PlexA Δcyto)^23^ also resulted in wing abnormalities following wounding (Fig. 3a). This indicates that PlexA Δcyto likely functions in a dominant-negative capacity by competing for the ligands of endogenous PlexA and that PlexA-mediated intracellular signalling is likely important for tissue repair. Interestingly, *Mical* inhibition did not cause a significant disruption of adult wing morphology following wounding, even though *Mical* knockdown by RNAi was efficient (Fig. 3a and Supplementary Fig. 2c). Taken together, these findings suggest that PlexA functions together with Sem**a**1a and Sema1b in tissue repair.

Vertebrate plexins function as a GTPase-activating protein (GAP) for the small GTPase Rap, thereby reducing the levels of active (GTP-bound) Rap. Semaphorin binding is predicted to increase the GAP activity of plexins through their dimerization, thereby reducing Rap activity^24^,^25^. In Drosophila, mutations in the *Rap1* gene disrupt morphogenesis in the embryo^26^. Rap1 regulates the stability of adherens junctions^27^ and decreasing the levels of Rap-GTP by expression of a GAP, Rapgap1, has been shown to increase the mobility of adherens jnctions by reducing their coupling to the actin cytoskeleton^28^. Thus, an attractive hypothesis is that the activation of PlexA signalling in damaged tissue increases GAP activity for Rap1 and thus allows cell rearrangements and tissue remodelling.

*Drosophila* has two rap-family GTPases, Rap1 and Rap2l, which are orthologues of vertebrate Rap1 and Rap2, respectively. In at least some situations, such as endothelial cell adhesion in mammals, Rap1 and Rap2 function antagonistically^29^. Moreover, mammalian plexins have a higher GAP activity for Rap1 (ref. 24). We therefore examined the effect of reducing the levels of both Rap1 and Rap2l. Reducing the levels of *Rap1* caused developmental defects even in the absence of injury (Fig. 3a), consistent with its role in embryonic morphogenesis and adherens junction formation^26^,^27^. In contrast, inhibition of *Rap2l* caused injury-dependent phenotypic abnormalities that were similar to those elicited by reducing *PlexA* levels (Fig. 3a,b). This is consistent with the possibility that both Rap2l and PlexA antagonize Rap1 function.

To further characterize the relationship between *PlexA* and the rap genes, we manipulated activity of the rap GTPases under conditions of *PlexA* overexpression. When *PlexA* was overexpressed, the adult wings showed blistering (Fig. 3c,d), suggesting a lack of proper adhesion between the cells on the dorsal and ventral surfaces. The blistering phenotype caused by *PlexA* overexpression was suppressed efficiently by knockdown of either *Rap2l* or *Mical* (Fig. 3c,d). In contrast, reducing Rap1 levels while overexpressing *PlexA* resulted either in a complete absence of wings or in lethality (Fig. 3c). Since the blistering phenotype elicited by overexpression of *PlexA* alone can be changed to phenotypes of no wings or lethality simply by increasing expression levels (Supplementary Fig. 2e), we conclude that Rap1 reduction likely enhances the effect of PlexA overexpression. If, as in mammals, PlexA reduces the activity of the rap GTPases in *Drosophila*, reducing Rap levels should enhance the *PlexA* overexpression phenotype. Indeed, we observed this kind of interaction with *Rap1* but not with *Rap2I*. Thus, our genetic data are consistent with PlexA functioning primarily as a GAP for Rap1 in *Drosophila* tissues rather than for Rap2l.

To test whether *Drosophila* PlexA possesses bona fide GAP activity for Rap GTPases, we performed an *in vitro* GAP assay. We purified the cytoplasmic domain of *Drosophila* PlexA connected to the GCN4 coiled-coil domain to promote its dimerization and performed a photometric GAP assay using purified *Drosophila* Rap1 and Rap2l as substrates, as previously described^24^. We found that PlexA catalyses GTP hydrolysis of rap GTPases, possessing about ninefold higher activity towards Rap1 compared with Rap2l (Fig. 3e,f), which is consistent with the predictions made by the genetic interactions.

As mentioned above, mammalian Rap1 and Rap2 have antagonistic roles in the context of endothelial cell adhesion; Rap1 promotes adhesion while Rap2 reduces adhesion^29^. Since knockdown of either *PlexA* or *Rap2l*, each of which can antagonize Rap1, impairs wound healing, the healing defect might be caused by a failure to downregulate Rap1 activity. Consist with this possibility, expression of a constitutively active Rap1 (V12) impaired wound repair (Fig. 3a). Further, the wing-defect phenotype caused by Rap1 knockdown was similarly suppressed by inhibition of PlexA or Rap2l, which would be predicted to restore Rap1 activity to some extent (Fig. 3g). Thus, taking our genetic and biochemical data together, we propose that PlexA regulates epithelial wound repair by functioning as a GAP primarily for Rap1. Since PlexA demonstrates GAP activities towards both Rap1 and Rap2l but with a higher activity for Rap1 *in vitro*, Rap1 could potentially suppress Rap2l regulation by PlexA in cells. Although Mical knockdown suppresses the *PlexA* overexpression phenotype, the absence of a healing defect with *Mical* knockdown might imply that Mical functions as an effector for PlexA only in a subset of functions or that the function of Mical is redundant in some contexts. Indeed, *C. elegans* does not have a recognizable *Mical* orthologue despite a clear role for semaphorin-plexin signalling in axonal pathfinding^30^.

### PlexA regulates delamination of epithelial cells

**Figure 4:**
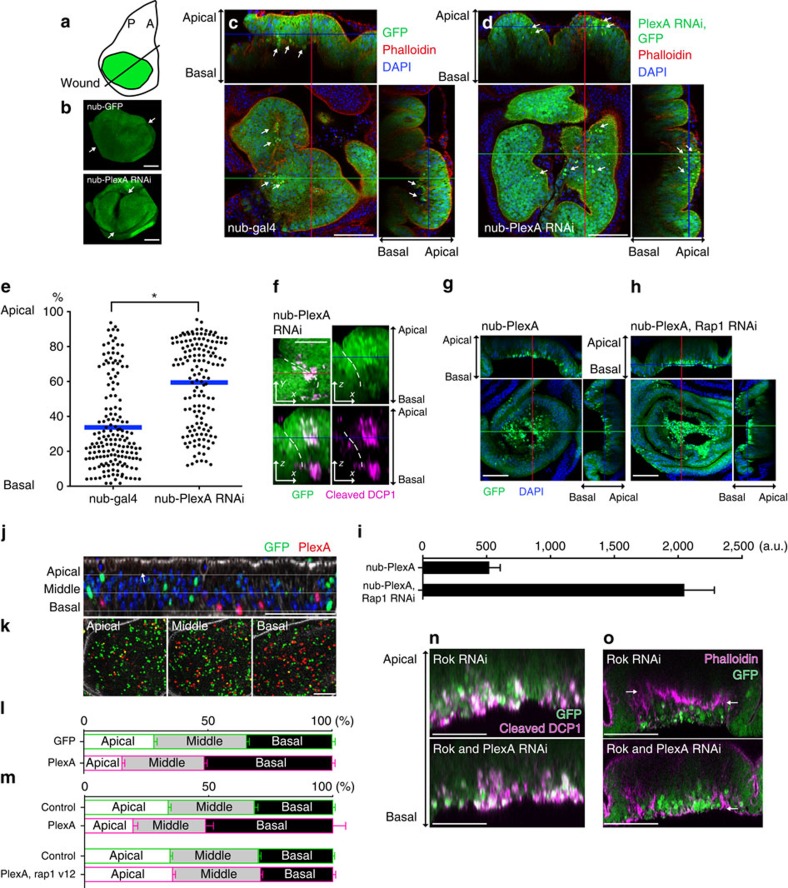
PlexA regulates delamination of epithelial cells. (**a**) Diagram of a bisected wing disc (A, anterior; P, posterior). (**b**) 3D reconstruction of wounded wing discs at 18 h.p.w. (Supplementary Movie 4). White arrows indicate the wound sites. (**c**,**d**) Control discs have many basally extruded cells at18 h.p.w. In *PlexA RNAi* discs, unextruded round cells with intense GFP signals are observed in the middle of the epithelial layer at 18 h.p.w. White arrows indicate examples of round cells with intense GFP fluorescence. (**e**) *z* axis location of the round cells with intense GFP fluorescence in damaged wing discs at 18 h.p.w. *P<0.05; two-tailed Mann–Whitney *U*-test. (**f**) The small cells with intense GFP signals in the middle of the epithelial layer contain the activated effector caspase DCP-1. Dotted lines indicate the wound edge. (**g**,**h**) Ectopic expression of *PlexA* induces basal cell extrusion, which is enhanced by concomitant inhibition of Rap1. (**i**) Quantification of basally extruded cells. Error bars represent s.e.m. (nub-PlexA: *n*=6, nub-PlexA, Rap1 RNAi: *n*=6). (**j**–**l**) *PlexA* expression in single-cell clones. Cells expressing PlexA also express RFP. Control cells express GFP alone. *PlexA* expression displaces cells to the basal side cell autonomously. Error bars represent s.e.m. (*n*=17 discs). (**m**) Basal displacement of cells by *PlexA* expression in single-cell clones is suppressed by Rap1 activation. Error bars represent s.e.m. (top: *n*=5, bottom: *n*=6). (**n**) *Rok RNAi*-mediated apoptosis is not affected by *PlexA* inhibition. (**o**) *Rok RNAi* induces cell delamination, with F-actin separating the epithelium from the basally extruded cells. PlexA inhibition perturbs *Rok RNAi*-induced cell delamination. White arrows indicate the plane of F-actin accumulation. Scale bars, 50 μm.

To examine the role of PlexA in imaginal discs, we first generated *PlexA*^*RNAi*^-expressing clones in undamaged discs. These clones were indistinguishable from control clones, indicating that mutant cells proliferate normally (Supplementary Fig. 3a,b). When we compared damaged discs expressing *PlexA*^*RNAi*^ with wild-type discs, the initial fusion of the two fragments, F-actin accumulation at the wound edge and JNK activation were indistinguishable from wounded wild-type discs (Supplementary Fig. 3c–e). In contrast, inhibition of JNK function interferes with these early events in wound healing^15^. However, unlike wild-type discs, healing *PlexA*^*RNAi*^ discs had a large cleft when visualized from the side of the peripodial epithelium (Fig. 4a,b and Supplementary Movie 4). In addition, wild-type discs had many small-volume cells with intense GFP fluorescence that resembled apoptotic cells basal to the epithelial layer. In *nub>PlexA*^*RNAi*^ discs some of these cells had been basally extruded, while others were observed throughout the thickness of the epithelium (Fig. 4c–e). These cells contained activated caspase, indicating that they were undergoing apoptosis (Fig. 4f). Thus, *PlexA* function appears necessary for efficient extrusion of dying cells.

To test whether increasing PlexA activity is sufficient to promote cell extrusion, we overexpressed *PlexA* in undamaged discs and observed extensive basal extrusion of cells (Fig. 4g). Most of these cells did not express activated caspase (Supplementary Fig. 3f,g), indicating either that PlexA promotes the extrusion of live cells or that the levels of caspase activation are below the threshold of detection by this method. To test whether PlexA functions in the cells being extruded or in their neighbours, we expressed *PlexA* in scattered single cells. *PlexA*-overexpressing cells were preferentially dislocated towards the basal side of the epithelium (Fig. 4j–l), indicating a cell-autonomous role of *PlexA* in this process. Dislocation of cells to the basal side by ectopic PlexA expression was enhanced by Rap1 inhibition and suppressed by Rap1 activation, consistent with the notion that PlexA promotes cell extrusion by reducing Rap1 activity (Fig. 4h,i,m). Furthermore, blocking apoptosis in damaged discs by expression of the baculoviral anti-apoptotic protein, p35, resulted in an increased number of adult wings with morphological defects upon wounding (Supplementary Fig. 3h,i). Thus, the death of damaged cells, as well as their efficient extrusion basally, might be a prerequisite for efficient repair of damaged epithelia with *PlexA* having a role in cell extrusion. This is consistent with the reported necessity for apoptotic cell death in damaged tissue before efficient regeneration in *Hydra* and in tadpole tails^31^,^32^.

To further explore the role of *PlexA* in basal extrusion of cells, we examined its requirement in a different situation. Inhibition of Rho kinase (Rok) results in misorientation of mitotic spindles causing cells to delaminate basally from the epithelium and subsequently undergo apoptosis^33^. In these discs, a condensation of F-actin is seen in the basal portion of the epithelium that separates the epithelium from the basally extruded cells (Fig. 4n). Concurrent expression of *PlexA*^*RNAi*^ does not prevent either apoptosis or the basal displacement of these cells, but it results in the retention of the delaminating cells within the basal portion of the epithelium rather than in their extrusion (Fig. 4o). This indicates that PlexA is also important for the epithelial remodelling induced by Rok inhibition.

### PlexA mediates uncoupling of F-actin and adherens junctions

**Figure 5:**
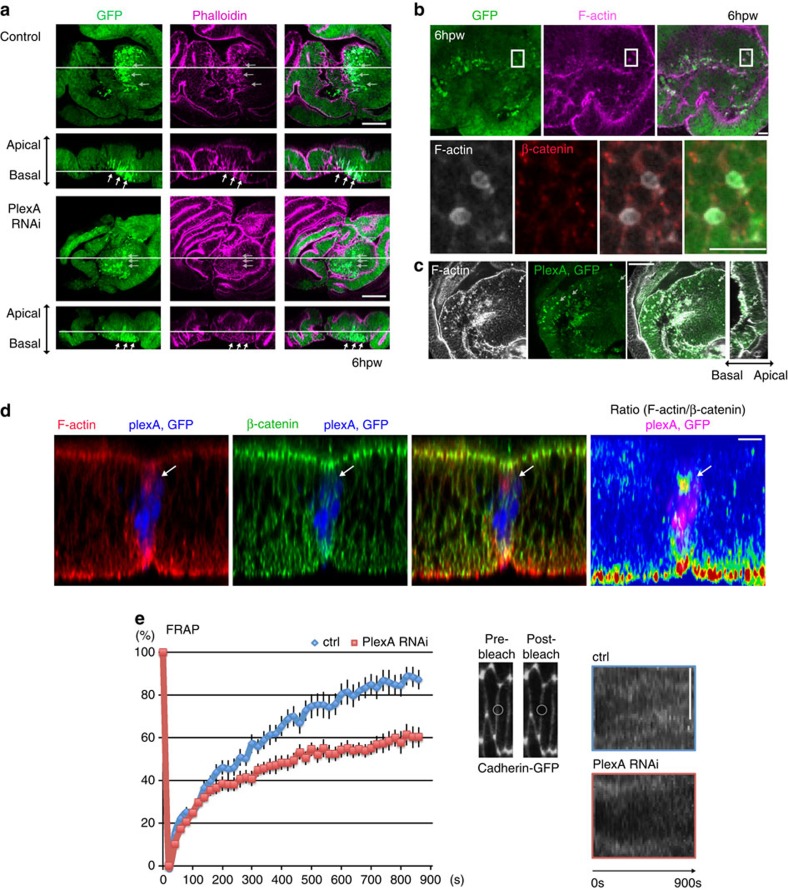
PlexA mediates uncoupling of F-actin and adherens junctions. (**a**) In control discs the round cells with intense GFP signals have pronounced accumulation of F-actin at 6 h.p.w. In *PlexA* RNAi discs, these cells have less accumulation of F-actin. White arrows indicate examples of round cells with intense GFP fluorescence. Data are representative of more than 10 images. (**b**) The round cells with intense GFP signals have actin rings around them at 6 h.p.w. (**c**) Extruded cells by ectopic expression of *PlexA* have actin rings around them (indicated with white arrows). Data are representative of more than five images. (**d**) Ratiometric analysis shows that cells with elevated *PlexA* have more F-actin uncoupled with adherens junctions compared with the surrounding cells. (**e**) FRAP analysis of cadherin–GFP in the pupal notum at 13 APF. *PlexA* RNAi inhibits fluorescence recovery of cadherin–GFP after photobleaching compared with control. Error bars represent s.e.m. (ctrl: *n*=20, *PlexA* RNAi: *n*=18). Right pictures show the process of photobleaching and kymographic analyses. Scale bars, 50 μm (**a**,**c**), 10 μm (**b**,**d**) and 5 μm (**e**).

How does PlexA dislocate cells to the basal side of the epithelial layer? In addition to the continuous F-actin accumulation along the wound edge, we also observed sporadic foci of F-actin accumulation in the vicinity of the wound (Fig. 5a), especially around the dying cells, which are characterized by intense GFP fluorescence as mentioned previously (Fig. 4f). At higher magnification, each of the F-actin foci could be seen as a ring structure surrounding the cell (Fig. 5b). Such actin rings are typically observed around cells undergoing apoptosis and extrusion^34^. Upon *PlexA* inhibition, the F-actin accumulation associated with dying cells was significantly reduced (Fig. 5a). Conversely, ectopic expression of *PlexA* in the whole-wing disc gave rise to many extruded cells with actin rings (Fig. 5c). Further, ectopic expression of *PlexA* in multiple-cell or single-cell clones for a limited time also induced F-actin accumulation around cells, especially on their apical side (Fig. 5d and Supplementary Fig. 4a,b). Importantly, while F-actin accumulates in or immediately around cells that are being extruding following wounding or *PlexA* overexpression, the polymerized F-actin does not induce a concomitant accumulation of adherens junctions, which was analyzed by measuring the ratio of F-actin to β-catenin (Fig. 5b,d and Supplementary Fig. 4a). Since Rap1 strengthens the coupling of F-actin and adherens junctions^28^,^29^,^35^,^36^, the lack of coupled accumulation of these two proteins is suggestive of reduced Rap1 activity resulting from the ability of PlexA to function as a GAP for Rap1.

The finding that ectopic PlexA expression induces an uncoupling of F-actin and adherens junctions prompted us to investigate the role of endogenous PlexA in the dynamic remodelling of adherens junctions because Rap1-mediated coupling of F-actin and adherens junctions is thought to stabilize adherens junctions^28^,^29^,^35^,^36^. Since it was difficult to perform a detailed analysis of junction dynamics in the wounded wing disc, we once again used the pupal notum epithelium to visualize adherens junction dynamics. *PlexA* inhibition affects not only wound repair (Fig. 2a) in the notum but also its normal development. Reducing PlexA levels in the notum results in an increased width of the midline region separating the microchaetes from ∼10 to 20 μm (Supplementary Fig. 5a). This is the region where the two wing discs fuse with each other during normal pupal development, a process that bears at least some superficial similarities to the rejoining of fragmented discs. The analysis of the pupal notum showed that *PlexA* inhibition induces a cleft in the midline (Supplementary Fig. 5b), which is reminiscent of the healing defect of the damaged wing disc (Fig. 4b). Although cell extrusion is known to occur in the midline of the notum during development^37^,^38^, we did not see an obvious effect of *PlexA* RNAi in reducing cell extrusion (Supplementary Fig. 5c), suggesting that the extent to which PlexA mediates cell extrusion depends on its context. To examine the dynamics of adherens junctions, we performed a fluorescence recovery after photobleaching (FRAP) analysis of cadherin–GFP in the epithelium of the midline notum. *PlexA* RNAi slowed down FRAP of cadherin–GFP (Fig. 5e), indicating that cadherin is less dynamic and more stable in the *PlexA* RNAi epithelia. This suggests that PlexA functions to reduce the stability of adherens junctions, thereby allowing the movement of cells in the epithelium with respect to their neighbours. Such cellular movements may be necessary for the extrusion of damaged cells and the subsequent remodelling of the epithelium to enable the re-establishment of epithelial continuity following damage.

### Plexin A1 regulates wound repair in zebrafish fins

**Figure 6:**
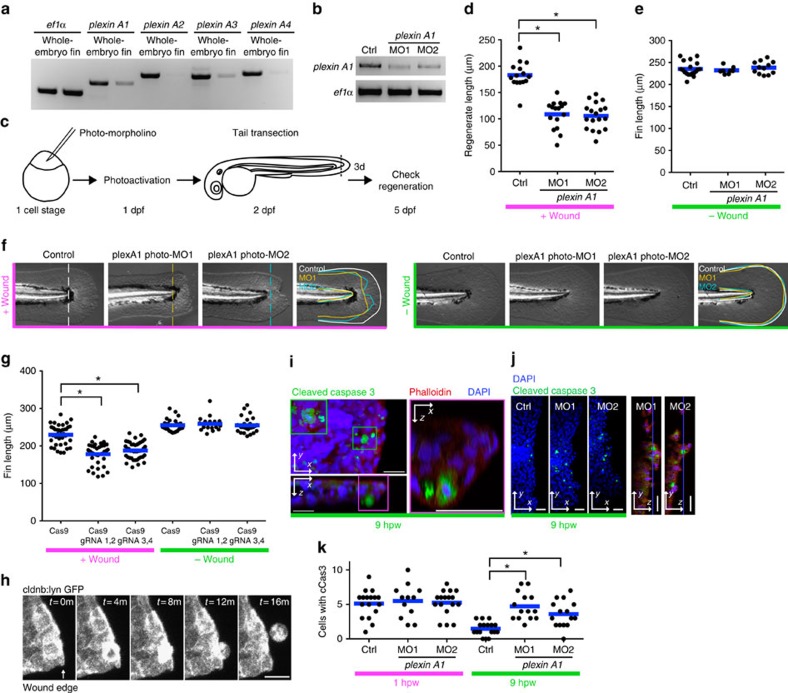
Plexin A1 regulates wound repair in zebrafish fins. (**a**) RT–PCR of plexins. *plexin A1* and *A3* are relatively enriched in tail fins at 2 d.p.f. (**b**) RT–PCR of *plexin A1* following knockdown. Two independent splice morpholinos were used. (**c**) Diagram of photo-morpholino injection, photoactivation and tail transection. (**d**) Regenerated fin length at 3 days post wounding (d.p.w.), i.e., 5 d.p.f. *P<0.05; one-way ANOVA with Dunnett's post-test. (**e**) Fin length at 5 d.p.f. in the absence of wounding. (**f**) Images of 5 d.p.f.-larval tail fins with/without wounding. Note that wounding affects both the length and width of the wounded tail fins in *plexin A1* morphants. (**g**) CRISPR-mediated *plexin A1* inhibition impairs fin regeneration at 3 d.p.w. (5 d.p.f.) but not the fin length without wounding. *P<0.05; one-way ANOVA with Dunnett's post-test. (**h**) Epithelial cell extrusion around a wound of *Tg(cldnb:lynGFP).* On average, 2.75 (*n*=4, s.e.m.=0.48) cells were observed to be extruded for 5 h imaging, but note that all the extrusion events were not captured because imaging was done only in a part of the tail fin. (**i**) An extruded cell is undergoing apoptosis. (**j**) Representative images of transected tail fins at 9 h.p.w. (**k**) Knockdown of Plexin A1 increases apoptotic cells at wounds at 9 h.p.w. but not 1 h.p.w. *P<0.05; one-way ANOVA with Dunnett's post-test.

Finally, to test whether plexins have a role in repairing damaged tissue in vertebrates, we utilized the well-characterized system of tail fin regeneration in zebrafish larvae, where defects in the earliest stages of wound repair impair fin regeneration at much later stages^2^. The zebrafish, *Danio rerio*, has four *PlexA* orthologues, *plexin A1, A2, A3* and *A4*. *plexin A1* and *A3* are expressed highly in larval tail fins (Fig. 6a). Reducing the levels of Plexin A3 using antisense mopholino oligonucleotides (MO) did not affect regeneration (Supplementary Fig. 6a–d). Reducing levels of Plexin A1 caused defects in early embryonic development. However, by using photo-morpholinos, which can be activated by ultraviolet light, we reduced Plexin A1 function 1 day post fertilization without significantly affecting normal development (Fig. 6b,c). Under these conditions, regeneration of the amputated tail fin was impaired, thus demonstrating a role for a *PlexA* orthologue in tissue repair in vertebrates (Fig. 6d–f). In addition to eliciting phenotypes using MOs, we also found that Plexin A1 inhibition through injection of Cas9 mRNA and two different gRNAs for *plexin A1* also impaired tissue repair (Fig. 6g).

We also examined earlier stages in wound healing by live imaging. During wound repair in tail fins, we observed epithelial cell extrusion around the wound edge (Fig. 6h and Supplementary Movie 5). Extruded cells had features of cells undergoing apoptosis (Fig. 6i). As wound healing proceeded, the number of apoptotic cells around wounds decreased in wild-type tail fins (Fig. 6j,k) but less so when the levels of Plexin A1 were reduced. Thus, consistent with our observations in *Drosophila*, reduction of Plexin A1 inhibited removal of the apoptotic cells around wounds in zebrafish. Although we have not defined the precise mechanism by which Plexin A1 regulates wound healing in zebrafish, these observations may reflect similar functions for plexins in wound repair in *Drosophila* and zebrafish.

## Discussion

We have described a role for *Drosophila* PlexA and for its zebrafish orthologue Plexin A1 in wound repair. PlexA exerts its function as a GAP for Rap1, which promotes coupling of F-actin and adherens junctions and thereby stabilizes adherens junctions^28^,^29^,^35^,^36^. On the basis of our data in *Drosophila*, we propose the following mechanism by which PlexA regulates epithelial wound repair (Supplementary Fig. 7a,b). Injury upregulates PlexA in cells in the vicinity of the wound (Fig. 1k), which leads to inhibition of Rap1 (Fig. 3f). PlexA-mediated Rap1 inhibition uncouples F-actin and adherens junctions, thus facilitating junctional remodelling (Figs 2 and 5). This is necessary for proper cell rearrangements including cell extrusion (Fig. 4), and the subsequent epithelial remodelling that occurs during wound repair. Since an actin regulator Mical functions downstream of PlexA in a context-dependent manner^23^ (Fig. 3c), it is possible that PlexA could also influence wound repair and epithelial remodelling through an additional mechanism such as Mical-mediated regulation of F-actin.

A recent study reported that PlexinB and its ligands Sema4b, 4d and 4g regulate renal wound repair and mitotoic spindle orientation in mammals^39^. We did not observe an obvious effect of PlexA inhibition either on mitotic orientation or mitosis in *Drosophila*. However, given that the experimental settings and the types of plexins and semaphorins differ between the two studies, it is possible that the other plexin and semaphorins have additional functions in mediating repair of *Drosophila* epithelia.

Finally, our findings are worth examining from an evolutionary perspective. Although we do not know whether the role of Plexin A1 in wound repair in zebrafish is mechanistically the same as that in *Drosophila* imaginal discs, our demonstration of a role for plexins in epithelial wound repair in metazoans that belong to two different superphyla (Ecdysozoa and Deuterostomia) suggests that this function could be evolutionarily ancient (Supplementary Fig. 7c). Plexins and semaphorins exist in all eumetazoa, in the placozoan *Trichoplax adhaerens* and in the sponge *Amphimedon queenslandica*. Thus, their origin predates the evolution of the nervous system. If the ancestral role for plexins is in wound repair, possibly in remodelling of adherens junctions and extruding damaged epithelial cells, it may have been subsequently modified in the nervous system to function in neurite repulsion. We therefore suggest that some of the other genes that have been studied primarily in the nervous system may also have a hitherto unsuspected role in epithelial wound repair.

## Methods

### *Drosophila* stocks

Flies were crossed and raised at 25 °C unless otherwise noted. For wild-type controls, Oregon-R (Bloomington stock centre (BL) 4269) was used unless otherwise specified. For the primary screening of CSS genes, RNAi stocks were collected from the BL and the Vienna *Drosophila* RNAi center (VDRC) based on a previous report^16^ and the LOCATE database (http://locate.imb.uq.edu.au). Several RNAis were also obtained from the National Institute of Genetics (NIG).

The following fly stocks were used in this study:

*UAS-dcr2; nub-gal4* (BL25754)

*UAS-GFP*

*UAS-his2A::RFP* (a gift from Dr Knoblich)

*UAS-GCaMP3* (BL32116)

*UAS-PlexA RNAi 1* (BL30483)

*UAS-PlexA RNAi 2* (VDRC4740)

*UAS-PlexA RNAi 3* (VDRC27240)

*UAS-PlexA RNAi 4* (VDRC107004)

*UAS-bsk DN (JNK DN)/TM6b*

*UAS-MICAL RNAi* (BL31148)

*UAS-Rap2l RNAi 1* (BL51840)

*UAS-Rap2l RNAi 2* (VDRC107745)

*UAS-Rap1 RNAi1* (BL29434)

*UAS-Rap1 RNAi2* (BL35047)

*UAS-Rap1 V12* (Hariharan lab, unpublished)

*UAS-sema1a RNAi 1* (BL29554)

*UAS-sema1a RNAi 2* (BL34320)

*UAS-sema1b RNAi 1* (BL28588)

*UAS-sema1b RNAi 2* (NIG6446R-3)

*UAS-HA-PlexA*^23^ (a gift from Dr Terman and Dr Kolodkin)

*UAS-HA-PlexAΔcyto*^23^ (a gift from Dr Terman)

*PlexA-myc BAC*^40^ (a gift from Dr Kolodkin)

*UAS-p35* (BL5073)

*UAS-Rok RNAi* (VDRC104675)

*UAS-dcr2; pnr-Gal4/TM3* (BL25758)

*UAS-dcr2; Act5c-Gal4/Cyo* (BL25708)

TRE reporter 12(a gift from Dr Bohmann)

*Sp; Dr / SM5-TM6B*

*UAS-mCherry-moesin/Cyo*

*Pnr-Gal4/TM3* (BL3039)

*E-Cad-GFP*

For the TIE-DYE experiment, the following stocks were used as previously described^41^.

*y, w, hsFLP; Sp; Dr / SM5-TM6b*

*w; Act < stop < lacz^nls^, Ubi < stop < GFP^nls^; Act < stop < gal4, UAS-his2A::RFP/SM5-TM6b*

Using above stocks, the following stable stocks were built and used for experiments.

*UAS-dcr2; nub-Gal4, UAS-GFP*

*UAS-dcr2; nub-Gal4, UAS-GFP, UAS-HA-PlexA*

*UAS-dcr2; nub-Gal4, UAS-GFP; UAS-PlexA RNAi 1*

*UAS-dcr2; nub-Gal4, UAS-GFP; UAS-PlexA RNAi 2*

*UAS-dcr2; nub-Gal4, UAS-GFP; UAS-sema1b RNAi 1*

*UAS-dcr2; nub-Gal4, UAS-GFP; UAS-sema1b RNAi 2*

*UAS-dcr2; nub-gal4, UAS-GFP; UAS-Rap2l RNAi 1*

*nub-Gal4; UAS-RFP*

*hsFLP;; UAS-PlexA RNAi 1*

*hsFLP; UAS-HA-PlexA*

*hsFLP; UAS-HA-PlexA; UAS-GFP, Act < stop < gal4, UAS-GFP*

*UAS-mCherry-Moesin, E-Cadherin–GFP/Cyo; Pnr-Gal4/TM6b*

### *In situ* wound of wing discs

L3 larvae of the *nub>GFP* stock were quickly immobilized in ice water, and the right wing pouch was bisected by applying pressure on the larval cuticle with a thin and dull-pointed tungsten needle with the help of GFP fluorescence (see Supplementary Movie 1). This procedure does not involve penetration of the larval cuticle. For each fly stock, at least 25 larvae were wounded.

### Calcium imaging

L3 larvae (*nub-gal4/UAS-GCaMP3; UAS-RFP/+*) were quickly immobilized in ice water and the right wing pouch was visualized using RFP and injured with a tungsten needle. Immediately after wounding, a larva was transferred on a slide glass and a coverslip was put on the larva to immobilize it completely. Live imaging of GCaMP3 fluorescence was performed with a stereomicroscope (Zeiss SteREO Lumar V12). Images were acquired every 10 s. For kymographic analysis of a calcium flash, signals in a rectangular box were summed into a 1D line and kymograph was made with ImageJ (NIH).

### Immunofluorescence and confocal imaging

Wing discs in late L3 larvae were dissected in PBS, fixed with paraformaldehyde in PBS and washed in 0.1% Triton in PBS. We used the following antibodies and fluorescent dyes: mouse 9E10 myc antibody (1:100, sc-40, Santa Cruz), rabbit cleaved *Drosophila* Dcp-1 antibody (1:400, #9578, Cell Signaling), mouse anti-Arm (1:100, N2 7A1, DHSB), 4,6-diamidino-2-phenylindole (DAPI; 1:1,000, Molecular Probes), rhodamine-conjugated phalloidin (1:500, Sigma-Aldrich) and Alexa Fluor 633 Phalloidin (1:50, Invitrogen). Fluorescence images were acquired with a confocal microscope (Zeiss LSM700). Three-dimensional reconstruction was performed by the Zen software. *z* axis analysis was performed using Volocity (PerkinElmer). For the TIE-DYE clone analysis in Supplementary Fig. 3a,b, GFP signals that overlap with RFP signals were subtracted from the GFP channel to make the analysis simple. After the subtraction, clone sizes were quantified using ImageJ. For FRAP experiments, fluorescence of cadherin–GFP in 2-μm-diameter spots were bleached in the epithelia of the midline notum at 13 after puparium formation (APF) and images were acquired every 20 s. Fluorescence recovery was quantified using ImageJ. For analysis of cell extrusion/delamination in the midline notum, time-lapse images with Ecad-GFP were acquired in the midline of the notum from 13 to 25 APF. *PlexA* RNAi 1 was expressed in the notum using *pnr-gal4*.

### Laser ablation and imaging

Flies with genotype w; *UAS-mCherry-Moesin, E-Cadherin–GFP*; pnr-GAL4/TM6b were crossed with *PlexA* RNAi 1 and kept at 29 °C. Pupae were staged at 13 h after puparium formation, and the notum epithelium was imaged at 29 °C on an Andor Revolution XD spinning disk imaging system (Nikon Eclipse Ti-E and Yokogawa CSU-x1) with a Nikon (Plan Apo VC PFS) × 60 oil-immersion objective. Pupae mounting was performed as previously described^11^. Wounds were made using a MicroPoint (Andor Technology) ultraviolet nitrogen-pumped dye laser (435 nm)—15 pulses at maximum power. Images were acquired before and immediately after wounding every 2.5 min for 6 h.

### Wound area and junction quantification in *Drosophila* pupae

Wound area over time was quantified using Fiji manual selection tool and area measure function. Junction ratio (total junction length/vertex distance) was obtained by dividing the total membrane length value with the distance in a straight line between each end of the corresponding membrane vertex as shown in Fig. 2d. Measurements were done using five independent pupae for both the control and *PlexA* RNAi. At least 10 membranes from each pupa were randomly sampled and measured using the Fiji line segment tool. Data were processed in Microsoft Excel and GraphPad Prism 6.0.

### RT–PCR (Drosophila)

Ten wing discs in late L3 larvae were dissected in PBS and snap-frozen at −80 °C. RNA was prepared using the RNeasy Mini Kit (QIAGEN) and one-step RT–PCR (QIAGEN) was performed. To check efficiency of RNAi-mediated knockdown, wing discs in L3 larvae (*UAS-Dcr2/+ or Y; Act5c-gal4/UAS-RNAi or UAS-Dcr2/+ or Y; Act5c-gal4/+; UAS-RNAi/+*) were dissected. Oligonucleotide sequences for RT–PCR were as follows:

*αtub84B* forward, 5′-CCTTGTCGCGTGTGAAACACTTCC-3′;

*αtub84B* reverse, 5′-GTTGGCCGCATCCTCCTTACC-3′;

*PlexA* forward, 5′-AAACATTCCATCAAGCCAAAGACCTCG-3′;

*PlexA* reverse, 5′-TTAAGAGGACTTCCTGCGCGGC-3′;

*PlexB* forward, 5′-AGACTTGGAAAAGATTCGCCGTCG-3′;

*PlexB* reverse, 5′-GCAATCACCATCTAGTGTAGCAGC-3′;

*sema1a* forward, 5′-ATACCAAAATGTGTCTCGTCAAGGGC-3′;

*sema1a* reverse, 5′-CTTAGGCTATCGTACTGCTCGGTC-3′;

*sema1b* forward, 5′-CGATGAGTTTGTTCTAGTTGGTGCC-3′;

*sema1b* reverse, 5′-CCTCCACATCTCGGCTGACTTC-3′;

*sema2a* forward, 5′-GCAGTTTCCACATTAACGAGATTCAGG-3′;

*sema2a* reverse, 5′-GACCCAGATTGGTGCCCACATAG-3′;

*sema2b* forward, 5′-TGCTGCCCATCATCTGCGCC-3′;

*sema2b* reverse, 5′-CGTTTACCCTTGGAAACACAATTCAGG-3′;

*sema5c* forward, 5′-CGACAAAATCAAGTGTGGATTTGCTGG-3′;

*sema5c* reverse, 5′-CACGCGTACAGCTGGTTCTCAC-3′;

*MICAL* forward, 5′-GACGCAGTGGAGACTTGCTGCC-3′;

*MICAL* reverse, 5′-GCAGATCTGGTCCAAATAGTTGAGCC-3′;

### Clone generation with heat shocks

Clones were induced by heat shocks in a 37-°C circulating water bath. For experiments to make clones composed of multiple cells (Fig. 5d and Supplementary Figs 3a,b and 4a), a heat shock of 10 min was performed at 48–72 h after egg lay and the discs were dissected from late L3 larvae. For experiments to make single-cell clones (Fig. 4j–m and Supplementary Fig. 4b), a heat shock of 14.5 min was performed at 100-h after egg lay and the discs were dissected 10–12 h after the heat shock.

### *In vitro* rap GAP assay

The active PlexA dimer construct was generated by fusing the GCN4 coiled-coil motif to the N terminus of the cytoplasmic region (residues 1,306–1,944) of *Drosophila* PlexA. The construct corresponds to the mouse CC(d)PlexinA1_cyto_ as described previously, which displayed the highest RapGAP activity among all the tested dimer constructs^25^. The fusion was subcloned into a modified pET28 vector (Novagen) containing an N-terminal tandem His_6_-SUMO tag^42^. *Drosophila* Rap1 and Rap2l (residues 2–166 and 2–167, respectively) were subcloned into a modified pET28 vector containing an N-terminal His_6_ tag and a cleavage site for the C3 protease of human rhinovirus^24^,^25^. Proteins were expressed in the bacterial strain BL21 (DE3) and purified as previously described^25^. Removal of the SUMO and His_6_ tags was achieved by addition of the Ulp1 and C3 protease, respectively. Rap1 and 2l were GTP-loaded before use in the activity assays. GTP loading was achieved by incubating Rap1 or 2l at a final concentration of 450 μM over night at 4 °C in a buffer containing 9 mM GTP, 3.4 mM EDTA, 10 mM Tris-HCl pH 8.0, 10% glycerol, 150 mM NaCl and 2 mM dithiothreitol. The exchange was quenched by the addition of MgCl_2_ to a final concentration of 20 mM. Removal of excess GTP was achieved by passing the mixtures over a size exclusion column (Superdex 75 10/300 GL, GE Healthcare). The GAP activity of the active dimer form of *Drosophila* PlexA_cyto_ towards *Drosophila* Rap1 and 2l was measured using the photometric assay as describe by Webb and Hunter with minor modifications^24^,^25^,^43^. Briefly, the release of inorganic phosphate upon GTP hydrolysis is coupled to the reaction catalysed by purine nucleoside phosphorylase, which converts 2-amino-6-mercapto-7-methylpurine ribonucleoside (MESG) to its guanine base form (2-amino-6-mercapto-7-methylpurine) and ribose-1-phosphate in the presence of phosphate. MESG has a *λ*_max_ at 320 nm, while the guanine base product has a *λ*_max_ at 360 nm. The reaction was monitored spectroscopically by following the absorbance decay at 320 nm and the increase at 360 nm at 27 °C. PlexinA and Rap were mixed to final concentrations of 20 and 100 μM, respectively, in a reaction buffer containing 8 U ml^−1^ PNP, 300 μM MESG, 20 mM Tris-HCl pH 7.6, 20 mM NaCl, 5% glycerol, 2 mM TCEP and 2 mM MgCl_2_. Each reaction was repeated three times. Owing to lack of the catalytic machinery, basal GTP hydrolysis of Rap1 and Rap2l in the absence of plexin is extremely slow and virtually undetectable under these conditions^24^,^25^. Slow increases of the absorbance at 360 nm in reactions of either Rap1 or Rap2l without plexin were produced partially by spontaneous conversion of MESG. The absorbance increase was faster in reactions with Rap2l, which was because of precipitation of the protein, as indicated by the simultaneous increase at 320 nm. To eliminate these artefacts, the plexin-catalysed rates were calculated by subtracting the slopes of the Rap-alone reactions from the slopes of the linear portions of the corresponding reactions in the presence of plexin.

### Conditional gene inactivation by tissue-specific Cas9 expression

To induce wing-specific gene knockout, we generated flies that simultaneously carried the 11F02-GAL4 driver transgene^44^, a UAS-Cas9 transgene and a double-gRNA transgene. The UAS-Cas9 transgene was generated in a custom UAS vector that has a 10 × UAS sequence, gypsy insulators, a phiC31 attB sequence and a *vermilion* transgene. It was integrated into the attP40-landing site by phiC31-mediated recombination^45^. Double-gRNA vectors were generated as described^46^. The gene-specific 20-bp sequences of gRNAs used in the present study are shown below for each target gene.

*plexA*: 5′-GAGCTGTAGTACGTTGTAGC-3′, 5′-AATGCGAGAACGAGTTAATG-3′

*e* (*ebony*): 5′-GCCATCGTGCTCTACACCTC-3′, 5′-GACTCTCAGGTGAAGATCCG-3′

*wg* (*wingless*): 5′-GAGAAGGAAACAGCGACGCC-3′, 5′-GGTGACCCACTCGATTGCCA-3′

*ct* (*cut*): 5′-GGACGAACTGCAGCGCTTGA-3′, 5′-GAGCAGTGTGGCCCAGCATC-3′

*cv* (*crossveinless*): 5′-GAAGAACTGCTCCTGCTGCA-3′, 5′-GCTGTCCAAGAAGTCCCACG-3′.

### Zebrafish maintenance and general procedures

Adult AB zebrafish and larvae were maintained according to the standard protocols^47^. For tail transection, 2–3 days post fertilization (d.p.f.) larvae were anaesthetized in E3 containing 0.2 mg ml^−1^ Tricaine (ethyl 3-aminobenzoate; Sigma-Aldrich) and the tail fin was resected with a razor blade^2^. Regenerate length was quantified by measuring the distance between the caudal tip of the notochord and the caudal tip of the tail fin at 3 days after wounding. For time-lapse imaging, tail fins of *Tg(cldnb:lynGFP)*^48^ were transected at 3 d.p.f. and time-lapse imaging was started immediately. Zebrafish immunostaining was performed as previously described^2^. A rabbit cleaved caspase-3 antibody (1:300, #9661, Cell Signaling) was used with DAPI (1:1,000, Molecular Probes) and rhodamine-conjugated phalloidin (1:500, Sigma-Aldrich).

### MO injection and RT–PCR

MO oligonucleotides (Gene Tools) in Danieau buffer (58 mM NaCl, 0.7 mM KCl, 0.4 mM MgSO_4_, 0.6 mM Ca(NO_3_)_2_ and 5.0 mM HEPES, pH 7.1–7.3) were injected (3 nl) into one-cell stage embryos. Danieau buffer was used as a control. For Plexin A3 knockdown, 500 μM *plexin A3* splice MO1 (5′-AGGCATGACATTGACTCACTTGTTA-3′) or 500 μM *plexin A3* splice MO2 (5′-ATCTGAATGGTGTCTTACCATATCC-3′) was used. For Plexin A1 knockdown, 150 μM *plexin A1* splice MO1 (5′-AGCAGATAATTCTCTTACCGAGATC-3′) or 150 μM *plexin A1* splice MO2 (5′-ACCAGTCGTTTGCTACCGTACCTCC-3′) was used. For ultraviolet-mediated Plexin A1 knockdown, combination of 471-μM *plexin A1* Photo-MO1 (5′-ATCTCGGTAAGPGAATTATCTGC-3′) and 429-μM *plexin A1* splice MO1 or combination of 471 μM *plexin A1* Photo-MO2 (5′-GGTACGGTAPCAAACGACTGG-3′) and 429-μM *plexin A1* splice MO2 was used. Photoactivation of Photo-MOs was performed with the Gene Tools UV light box (Gene Tools) in 1 d.p.f. embryos as described previously^49^. For morphotyping of the splicing MOs, RNA was prepared from 2.5 d.p.f. larvae using TRIZOL (Invitrogen), and one-step RT–PCR (QIAGEN) was performed.

Oligonucleotide sequences used for morphotyping RT–PCR were as follows:

*plexin A1* forward, 5′-GCTGCAGACTGATATCCATGAGC-3′;

*plexin A1* reverse, 5′-GTGACATCCTCGTCCTGGAG-3′;

*plexin A3* forward, 5′-TGAATGGTGTTCCTCCGCCG-3′;

*plexin A3* reverse, 5′-TTGGACTTCAGAGAAAGCTTCACG-3′.

For expression analysis of plexins in larval tail fins, tail fins of 50 larvae at 3 d.p.f. were resected, RNA was prepared using TRIZOL and one-step RT–PCR was performed. Oligonucleotide sequences for RT–PCR were as follows:

*ef1α* forward, 5′-TACGCCTGGGTGTTGGACAAA-3′;

*ef1α* reverse, 5′-TCTTCTTGATGTATCCGCTGA-3′;

*plexin A1* forward, 5′-GGAACGTGGCATCCCTCATC-3′;

*plexin A1* reverse, 5′-GTGACATCCTCGTCCTGGAG-3′;

*plexin A2* forward, 5′-GACTACTTTCCAACAATCTCCAGCCG-3′;

*plexin A2* reverse, 5′-CGCAGAAGCCATTATAGACATAGGAGG-3′;

*plexin A3* forward, 5′-GTAAACTGGTGGCGGATGAGG-3′;

*plexin A3* reverse, 5′-AACACCACCGAATGGTCTCCG-3′;

*plexin A4* forward, 5′-TTGTTCGTGGCCACAGCTGTTG-3′;

*plexin A4* reverse, 5′-GAGTGGTTCTTGTACACGTAGGC-3′.

### CRISPR-Cas9 in zebrafish

We designed single guide RNAs (sgRNAs) by seeking sequences corresponding to GGN_18_NGG on the sense or antisense strand of *plexin A1* DNA. To generate sgRNAs, we performed PCR with Phusion polymerase without a template^50^; a unique oligonucleotide encoding the T7 polymerase-binding site and the sgRNA target sequence GGN_18_N (CRISPRF=GAAATTAATACGACTCACTATA GGN_18_ GTTTTAGAGCTAGAAATAGC); and a common oligonucleotide encoding the remainder of the sgRNA sequence (sgRNAR=AAAAGCACCGACTCGGTGCCACTTTTTCAAGTTGATAACGGACTAGCCTTATTTTAACTTGCTATTTCTAGCTCTAAAAC). The DNA templates were used for *in vitro* transcription by T7 RNA polymerase (Ambion). Four unique sgRNAs were generated using the following CRISPRF oligonucleotides:

sgRNA 1, GAAATTAATACGACTCACTATAGG GAGATGGAGTACGCCACTGTTTTAGAGCTAGAAATAGC;

sgRNA 2, GAAATTAATACGA CTCACTATAGGTAGCAAACGACTGGTTTAGTTTTAGAG CTAGAAATAGC;

sgRNA 3, GAAATTAATACGACTCACTAT AGGACAGAACCAAAGCGAGAGGTTTTA GAGCTAGAAATAGC

sgRNA 4,

GAAATTAATACGACTCACTATAGGACCGATCGACTCCATCACGTTTTAGAGCTAGAAATAGC.

For making Cas9 RNA, pCS2-nCas9n (Addgene 47929)^51^ was linearized by Not1 and *in vitro* transcribed by SP6 RNA polymerase (Ambion). RNA of Cas9 (250 ng μl^−1^) and two sgRNAs (15 ng μl^−1^ each) in Danieau buffer were injected (3 nl) into one-cell stage embryos. Cas9 only was used as a control.

### Statistics

Figure 1i: *P<0.05, two-tailed *χ*^2^-test with the Bonferroni correction (RNAi), two-tailed *χ*^2^-test (CRISPR), ctrl: *n*=211, PlexA RNAi 1: *n*=90, PlexA RNAi 2: *n*=25, PlexA RNAi 3: *n*=20, PlexA RNAi 4: *n*=27, Cas9: *n*=98, PlexA CRISPR: *n*=39.

Figure 2a: shadowed areas represent s.d. WT: *n*=6, PlexA RNAi: *n*=3.

Figure 2g: error bars represent s.e.m. *P<0.05; Kruskal–Wallis test with Dunn's post-test, WT before wound: *n*=51, PlexA RNAi before wound: *n*=52, WT after wound: *n*=51, PlexA RNAi after wound: *n*=51.

Figure 3a: *P<0.05, two-tailed *χ*^2^-test with the Bonferroni correction, ctrl: *n*=176, PlexA RNAi 1: *n*=44, PlexA RNAi 2: *n*=44, sema1a RNAi 1, sema1b RNAi 1: *n*=88, sema1a RNAi 2, sema1b RNAi 2: *n*=53, PlexA Δcyto: *n*=85, MICAL RNAi: *n*=150, Rap2l RNAi 1: *n*=68, Rap2l RNAi 2: *n*=128, Rap1 V12: *n*=128, Rap1 RNAi 1: *n*=77, Rap1 RNAi 2: *n*=60.

Figure 4e: *P<0.05; two-tailed Mann–Whitney *U*-test, nub-gal4: 10 discs, 150 cells, nub-plexA RNAi: 10 discs, 166 cells.

Figure 4i: error bars represent s.e.m. (nub-PlexA: *n*=6, nub-PlexA, Rap1 RNAi: *n*=6).

Figure 4l: error bars represent s.e.m. (*n*=17 discs).

Figure 4m: error bars represent s.e.m. (top: *n*=5, bottom: *n*=6).

Figure 5e: error bars represent s.e.m. (ctrl: *n*=20, PlexA RNAi: *n*=18).

Figure 6d: *P<0.05; one-way analysis of variance (ANOVA) with Dunnett's post-test, Ctrl: 15 larvae, MO1: 16 larvae, MO2: 18 larvae.

Figure 6g: *P<0.05; one-way ANOVA with Dunnett's post-test, Cas9, +wound: 38 larvae, Cas9, gRNA 1,2, +wound: 38 larvae, Cas9, gRNA 3,4, +wound: 42 larvae, Cas9, −wound: 25 larvae, Cas9, gRNA 1,2, −wound: 17 larvae, Cas9, gRNA 3,4, −wound: 17 larvae.

Figure 6k: *P<0.05; one-way ANOVA with Dunnett's post-test, Ctrl 1 h.p.w.: 17 larvae, MO1: 1 h.p.w.: 12 larvae, MO2: 1 h.p.w.: 17 larvae, Ctrl 9 h.p.w.: 17 larvae, MO1: 9 h.p.w.: 14 larvae, MO2: 9 h.p.w.: 15 larvae.

Supplementary Fig. 3h: *P<0.05, two-tailed *χ*^2^-test, +wound/ctrl: *n*=99, +wound/p35: *n*=91, −wound/ctrl: *n*=573, −wound/p35: *n*=310.

Supplementary Fig. 5a: *P<0.05, two-tailed unpaired *t*-test, pnr-gal4: *n*=7, pnr-PlexA RNAi: *n*=6.

### Data availability

The data that support the findings and reagents used in this study are available from one of the corresponding authors (S.K.Y.) upon request.

## Additional information

**How to cite this article:** Yoo, S. K. *et al.* Plexins function in epithelial repair in both *Drosophila* and zebrafish. *Nat. Commun.* 7:12282 doi: 10.1038/ncomms12282 (2016).

## Supplementary Material

Supplementary Information Supplementary Figures 1-7

Supplementary Movie 1 In situ damage of wing discs in an L3 larva. A wing pouch expressing GFP (nubbin-GFP) was wounded with a tungsten needle while leaving the cuticle intact.

Supplementary Movie 2 Wounding induces a calcium flash in wing discs. Calcium imaging was performed in the wing pouch immediately after wounding (nub-Gal4/UASGCaMP3; UAS-RFP/+). Note that the calcium flash is rapid and transient, ceasing within 10 minutes after wounding.

Supplementary Movie 3 Laser wounding in the notum. Time-lapse imaging of wound closure in the notum of pupae, starting at 13h after puparium formation. PlexA inhibition impairs wound closure compared to control.

Supplementary Movie 4 3D reconstruction of wing pouches at 18 hours post wounding (left/control, right/PlexA RNAi). The control wing pouch looks grossly normal with extruded cells at the basal side at 18 hours post wounding (left). The PlexA-inhibited wing pouch has a cleft on the apical surface (right).

Supplementary Movie 5 Cell extrusion in a zebrafish wound. Time-lapse imaging of a wounded tail fin in Tg(cldnb:lynGFP) at 3 dpf. Extruded cells are indicated with an arrowhead.
