# Supplementary material for: Plexins function in epithelial repair in both Drosophila and zebrafish
Source: Nat Commun. 2016 Jul 25;7:12282. doi: 10.1038/ncomms12282 (PMC4962468; doi:10.1038/ncomms12282)
Supplement: Supplementary Information — Supplementary Figures 1-7 [file ncomms12282-s1.pdf]

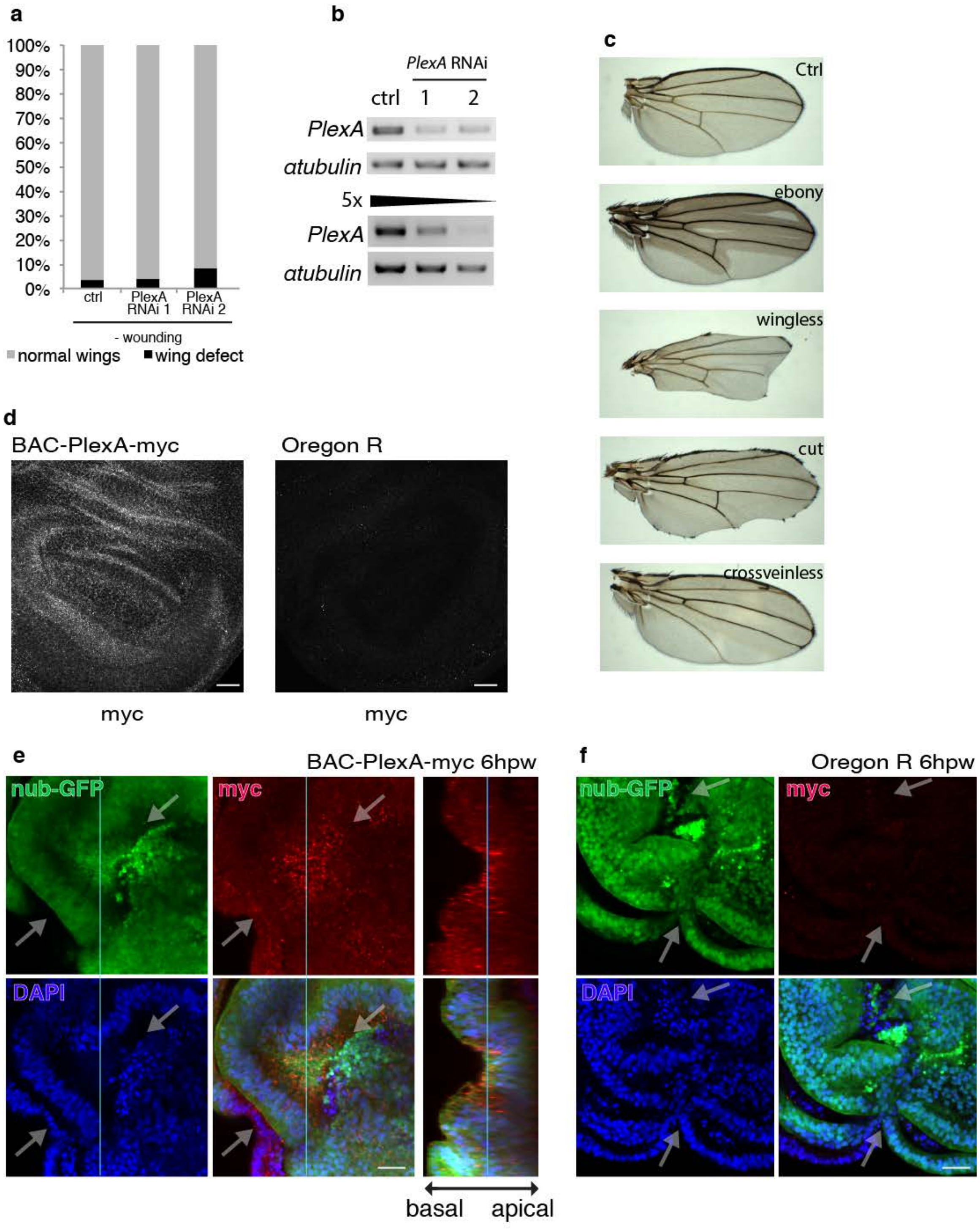

Supplementary Figure 1 *PlexA* expression in wing discs

a. Inhibition of *PlexA* by two different *PlexA* RNAi transgenes does not have a strong affect on adult wing morphology during normal development (ctrl: n=371, *PlexA* RNAi 1: n=194, *PlexA* RNAi 2: n=543).

b. Semi-quantitative RT-PCR of *PlexA* using RNA from wing discs. Two independent *PlexA* RNAi transgenes decrease the amount of *PlexA* mRNA. The two lower panels show that the semi-quantitative RT-PCR under this condition can detect a 5-fold difference in template levels.

c. Positive controls of the wing disc specific knockout with CRISPR. A stock with *11F02-Gal4* and *UAS-Cas9* was crossed with stocks with positive control gRNAs. Each wing exhibits the expected loss-of-function phenotype of *ebony*, *wingless*, *cut* or *crossveinless*.

d. Myc-immunofluorescence in wing discs of myc-epitope tagged *PlexA* (*PlexA-myc)* BAC transgenic and control larvae. An anti-myc antibody labels the wing discs of the *PlexA-myc* BAC transgenic, but not control larvae.

e. PlexA expression detected by myc immunofluorescence in *PlexA-myc* BAC transgenics is pronounced at the basal side of epithelial cells in the vicinity of the wound. Note that PlexA expression is not co-localized with cell debris with high GFP fluorescence, indicating that the increased expression of PlexA is not due to a nonspecific volume effect.

f. Myc staining in control flies. Negative control for the data in d.

Scale bars: 20 μm

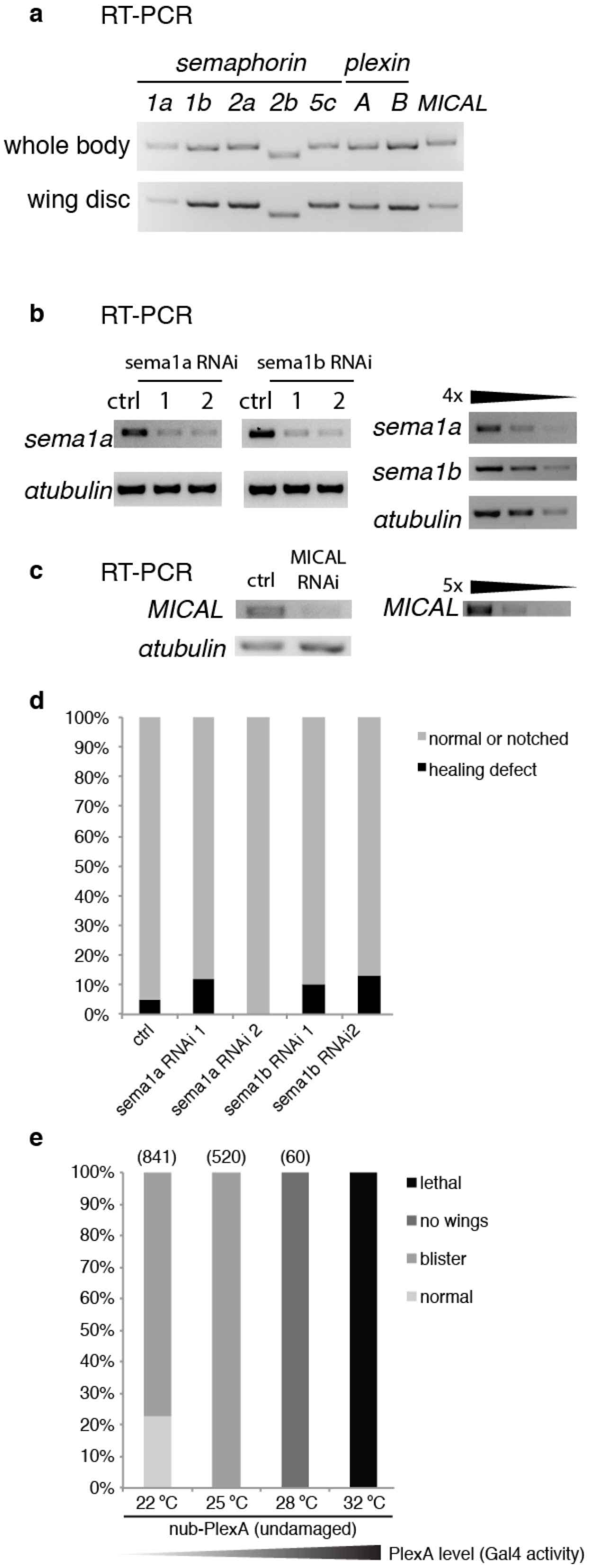
a
RT-PCR
semaphorin
plexin
1a 1b 2a 2b 5c A B MICAL
whole body
wing disc
b
RT-PCR
sema1a RNAi
sema1b RNAi
ctrl 1 2 ctrl 1 2
sema1a
atubulin
4x
sema1a
sema1b
atubulin
c
RT-PCR
ctrl
MICAL RNAi
MICAL
atubulin
5x
MICAL
d
100%
90%
80%
70%
60%
50%
40%
30%
20%
10%
0%
normal or notched
healing defect
ctrl
sema1a RNAi 1
sema1a RNAi 2
sema1b RNAi 1
sema1b RNAi2
e
(841)
(520)
(60)
lethal
no wings
blister
normal
22 °C
25 °C
28 °C
32 °C
nub-PlexA (undamaged)
PlexA level (Gal4 activity)

Supplementary Figure 2 Sema-PlexA signaling in wing discs

a. RT-PCR of genes encoding Sema-PlexA signaling components.

b. Semi-quantitative RT-PCR of *sema1a* and *sema1b* using RNA from wing discs. Two independent *sema1a* or *sema1b* RNAi's decrease the amount of their respective transcripts. The panels on the right show that the semi-quantitative RT-PCR under this condition can detect a 4-fold difference of template amounts.

c. Semi-quantitative RT-PCR of *MICAL* using RNA from wing discs. A *MICAL* RNAi reduces transcript levels effectively. The panels on the right show that the semi-quantitative RT-PCR under this condition can detect a 5-fold difference of template amounts.

d. Single knockdown of either *sema1a* or *sema1b* has little effect on wound repair (ctrl: n=53, *sema1a* RNAi 1: n=65, *sema1a* RNAi 2: n=70, *sema1b* RNAi 1: n=85, *sema1b* RNAi 2: n=51).

e. The phenotypes induced by ectopic expression of PlexA in the wing pouch differ depending on its expression levels. Note that higher temperatures result in higher expression of PlexA due to the temperature dependency of the Gal4/UAS system.

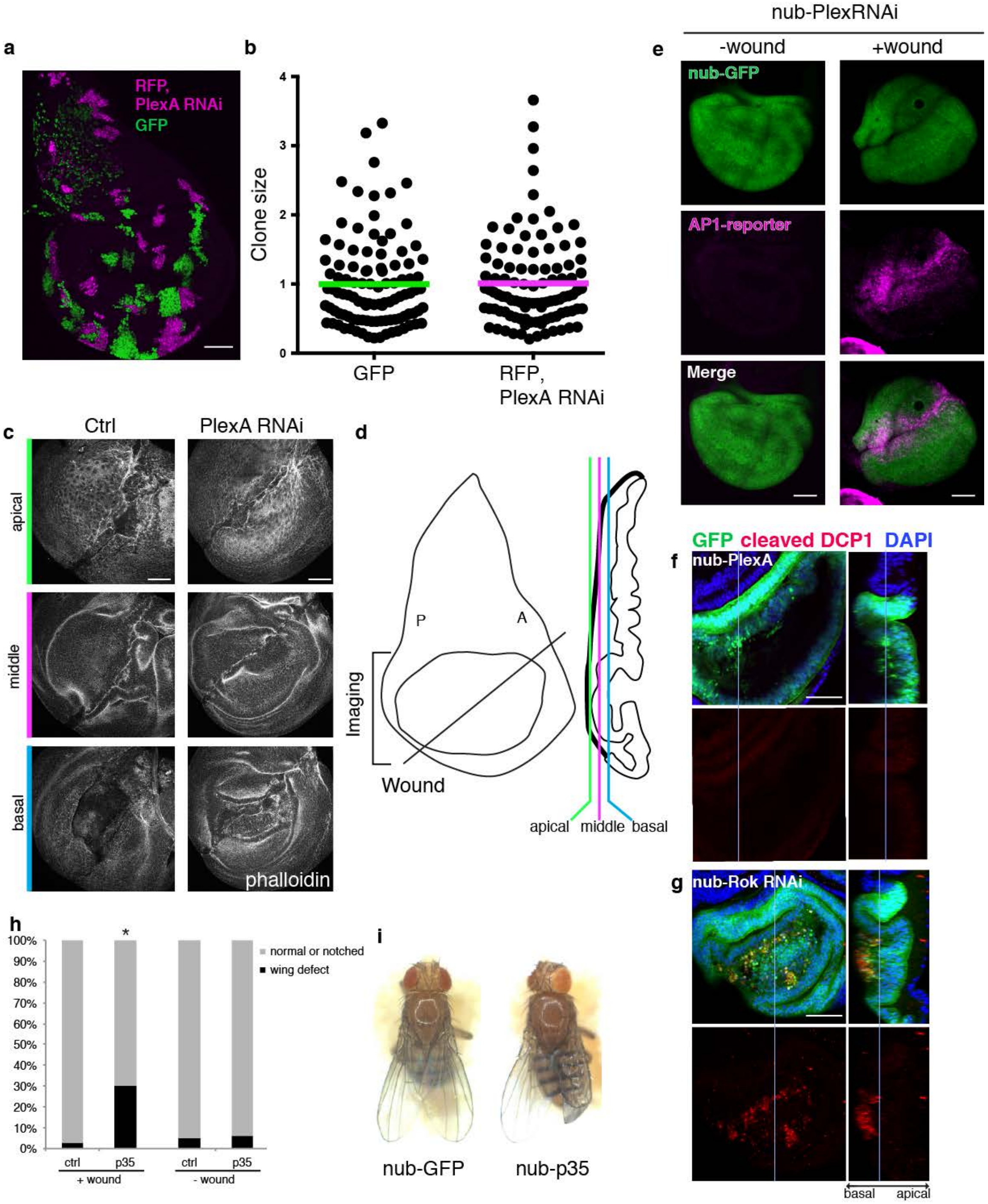

Supplementary Figure 3 Wing disc analysis

a. Clones generated using the TIE-DYE system (*hsFLP/+; ubi<stop<GFP/+; act5c<stop<gal4, UAS-his2A::RFP/UAS-PlexA RNAi*). *PlexA* knockdown does not affect clone morphology, size or boundary shape.

b. Size quantification of clones generated by TIE-DYE (6 discs, GFP: 104 clones; RFP, *PlexA* RNAi: 100 clones).
c. *PlexA* RNAi does not affect either fusion of bisected wing discs or F-actin accumulation at the wound edges at 6 hpw.
d. Diagram of Z-section planes.
e. *PlexA* RNAi does not affect JNK activation at the wound edge at 6 hpw.
f. Ectopic expression of *PlexA* induces basal cell extrusion but not apoptosis.
g. Compared to *PlexA*-induced cell extrusion, knockdown of *Rok* by *Rok* RNAi induces both basal cell extrusion and apoptosis.
h. Expression of p35 in the wing pouch perturbs epithelial repair upon wounding, but not normal wing development without wounding (+wound/ctrl: n=99, +wound/p35: n=91, -wound/ctrl: n=573, -wound/p35: n=310). *, $p < 0.05$, two-tailed Chi-square test.
i. Representative pictures of adult wings. Note that only right wing discs are wounded in L3 larvae.

Scale bars: 50 μm

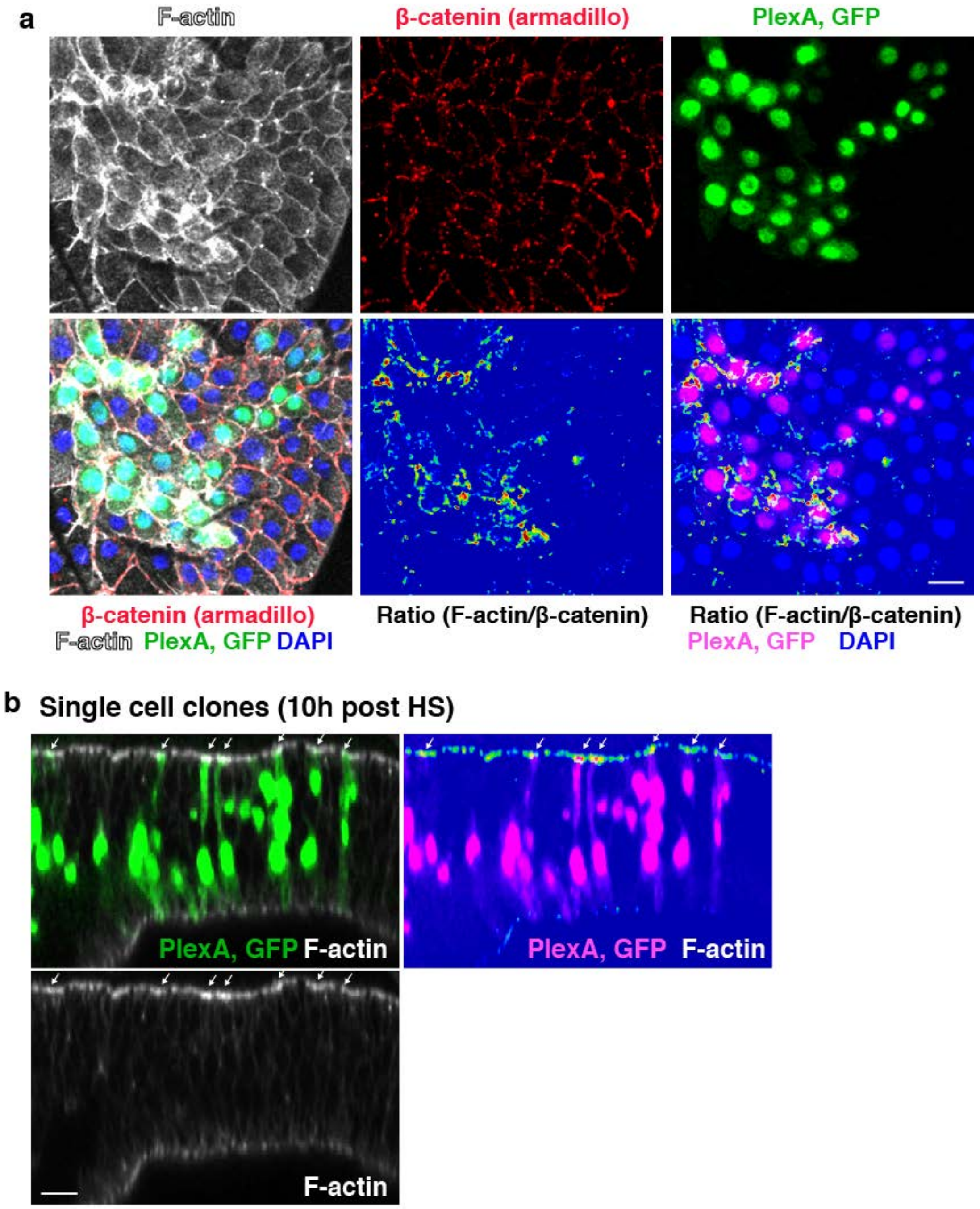

Supplementary Figure 4 PlexA mediates uncoupling of F-actin and adherens junctions.

a. PlexA expression in the peripodial epithelium of the wing disc induces accumulation of F-actin but not β-catenin.

b. PlexA expression in single cell clones in the disc proper of the wing disc induces F-actin accumulation at the apical regions of cells.

Scale bars: 10 μm

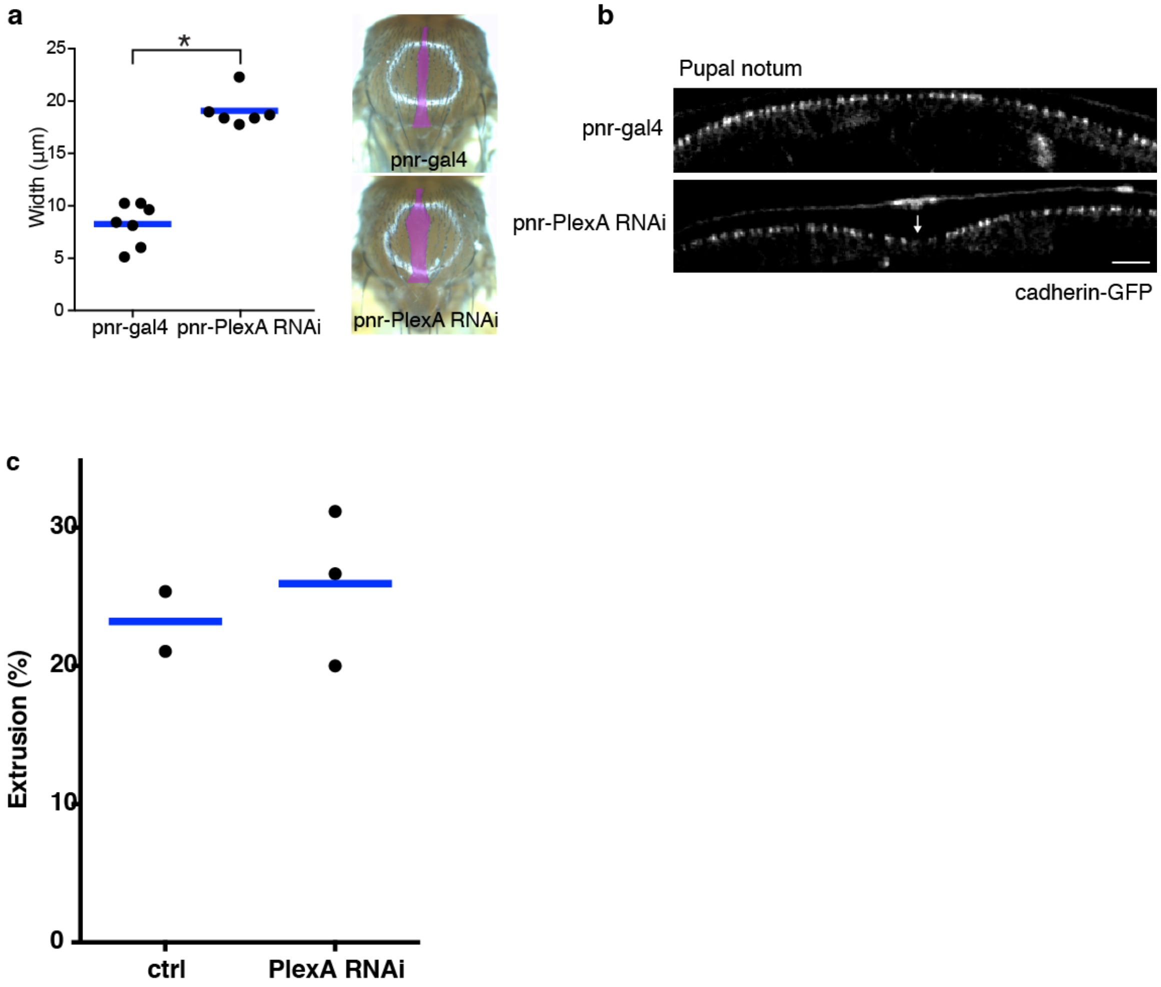

Supplementary Figure 5 PlexA regulates the developmental process of the notum epithelia.

a. Inhibition of PlexA in the midline of the notum makes the width between central microchaetae wider. *, $p < 0.05$, two-tailed unpaired t-test.
b. Inhibition of PlexA in the middle of the notum induces a cleft in pupae at 24 APF (after puparium formation).
c. Cell extrusion/delamination events were imaged in the midline of the notum from 13 APF to 25 APF. The percentage of cells delaminating in the midline was calculated as the ratio between the number of cells that delaminated in 12 hours and the total initial number of cells.

Scale bar: 20 μm

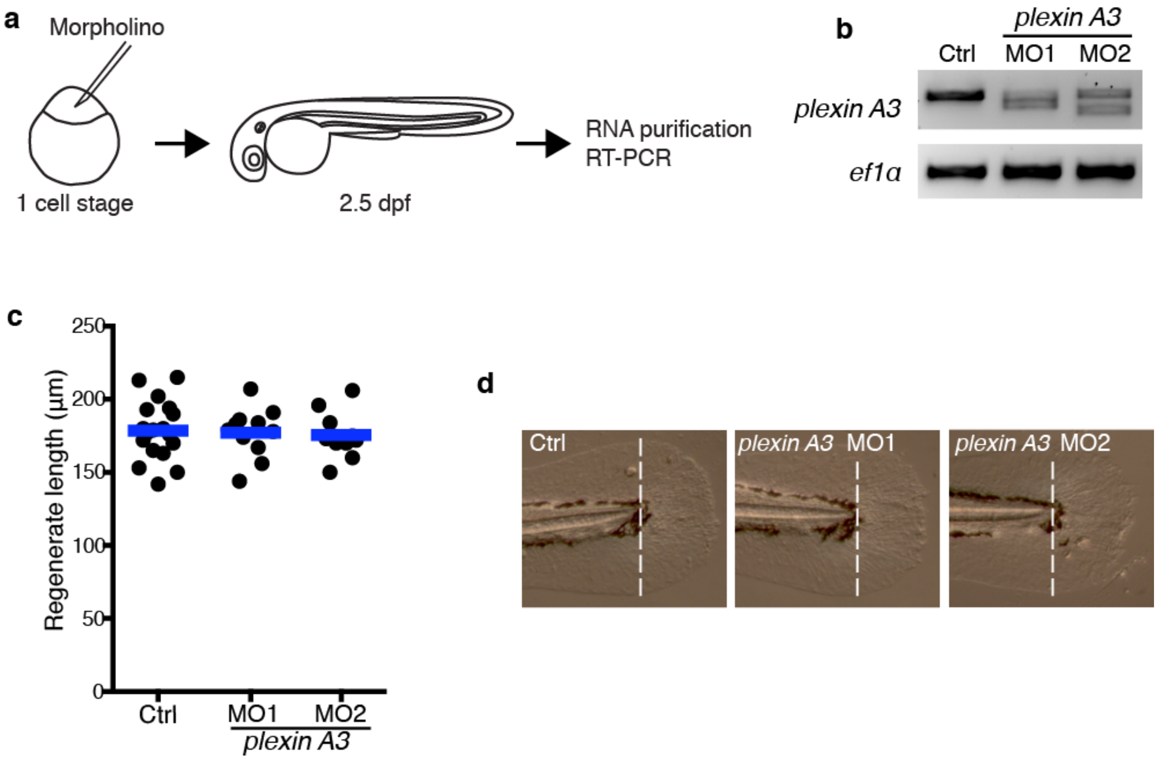

Supplementary Figure 6 Plexin A3 in zebrafish

a. Schematic of morpholino injection and RNA purification.
b. RT-PCR of plexinA3 with/without MOs. Two different splice morpholinos were used.
c. Quantification of regenerated fin length at 3 dpw (5 dpf) (Ctrl: 18 larvae, MO1: 11 larvae, MO2: 10 larvae).
d. Representative pictures of regenerated fins at 3 dpw (5 dpf).

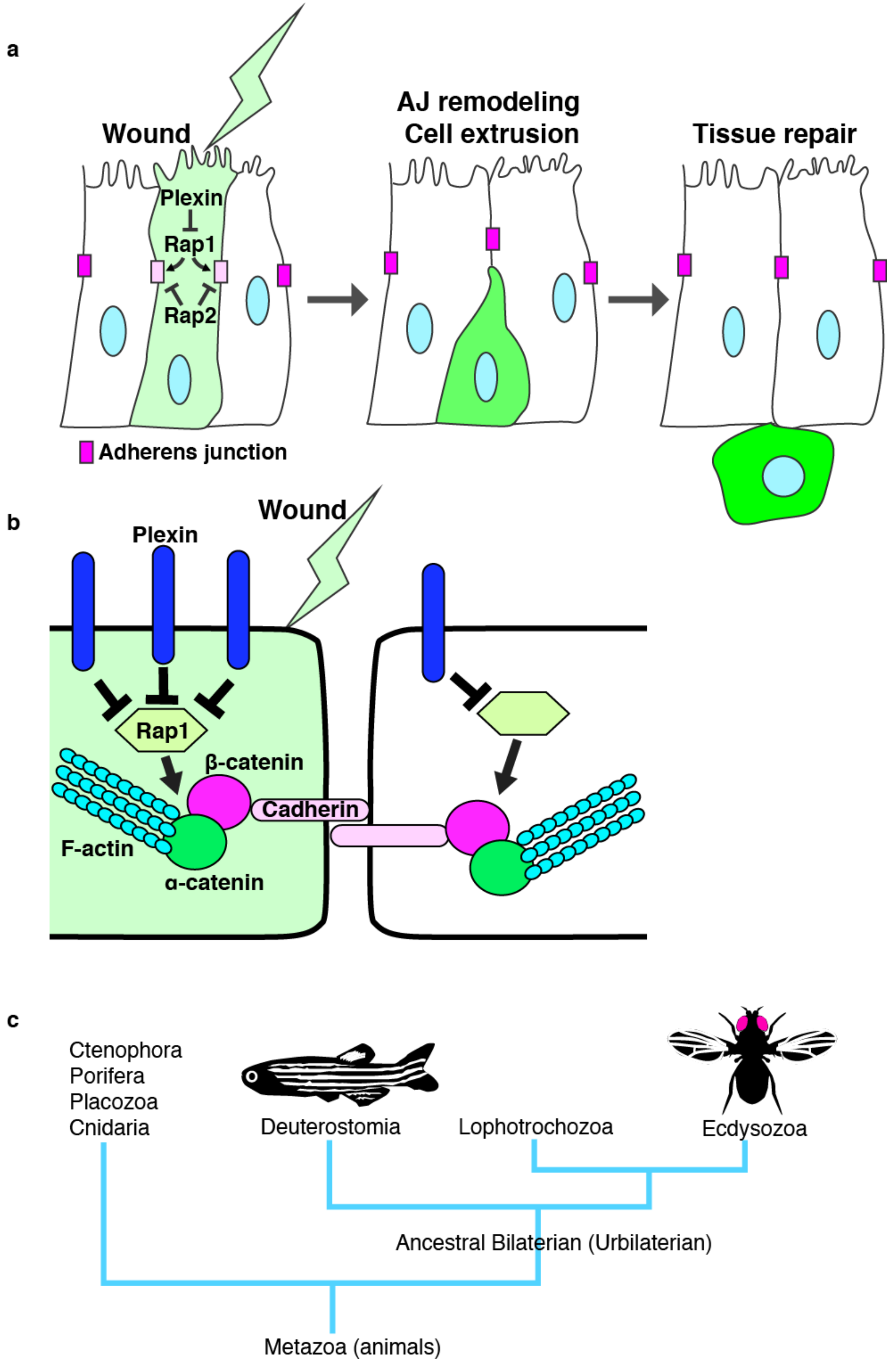
a
Wound
Plexin
Rap1
Rap2
AJ remodeling
Cell extrusion
Tissue repair
Adherens junction
b
Wound
Plexin
Rap1
β-catenin
Cadherin
F-actin
α-catenin
c
Ctenophora
Porifera
Placozoa
Cnidaria
Deuterostomia
Lophotrochozoa
Ecdysozoa
Ancestral Bilaterian (Urbilaterian)
Metazoa (animals)

Supplementary Figure 7 An evolutionarily ancient role for plexins in cell rearrangement during epithelial repair

a. A schematic of the model that we propose. Wounding activates plexin signaling, leading to Rap1 inhibition. This results in disruption of Rap1-mediated coupling of F-actin and adherens junctions and induces cell rearrangement and extrusion. We propose that these processes are critical for efficient wound repair.
b. A detailed schematic of PlexA-Rap1-mediated remodeling of adherens junctions.
c. A phylogenetic tree of Metazoa. Zebrafish belong to Deuterostomia and *Drosophila* belongs to Ecdysozoa.
